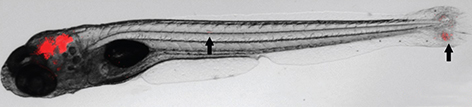# CXCR3-CXCL11 axis: recruiting macrophages in mycobacterial infection

**Published:** 2015-03

During infection, mycobacteria exploit the immune system and parasitise macrophages in order to disseminate inside the host. Macrophage recruitment to the site of infection is regulated by chemokine release and signalling, and CXC chemokine receptor 3 (CXCR3) is particularly important for directing macrophage activity. However, its role in mycobacterial disease is still poorly understood. To elucidate this, Annemarie H. Meijer and colleagues used *Mycobacterium marinum*-infected zebrafish embryos, a well-established model of tuberculosis, and knocked out a zebrafish orthologue of *CXCR3*, *cxcr3.2*. They found that, in zebrafish mutants, both infection-dependent recruitment of macrophages and mycobacterial dissemination are attenuated. Notably, this effect can be mimicked by treatment with the CXCR3 antagonist NBI74330. In addition, the authors identified two homologues of the human chemokine (C-X-C motif) ligand 11 (CXCL11) family that are inducible by infection and able to recruit macrophages via Cxcr3.2 activation. These results point to the CXCR3-CXCL11 axis as a new potential target for anti-tuberculosis therapy.

**Page 253**

**Figure:**